# Plasmalogen deficiency and the Alzheimer’s disease risk of apolipoprotein E4

**DOI:** 10.1093/braincomms/fcag040

**Published:** 2026-02-12

**Authors:** Jenny Hällqvist, Jan-Willem Taanman, Andreas Göteson, Wendy E Heywood, Jonathan M Schott, John Hardy, Mikael Landén, Henrik Zetterberg, Kevin Mills, Lionel Ginsberg

**Affiliations:** Translational Mass Spectrometry Research Group, UCL Great Ormond Street Institute of Child Health, London WC1N 1EH, UK; Department of Clinical and Movement Neurosciences, UCL Queen Square Institute of Neurology, London NW3 2PF, UK; Department of Psychiatry and Neurochemistry, Institute of Neuroscience and Physiology, University of Gothenburg, Gothenburg 431 41, Sweden; Translational Mass Spectrometry Research Group, UCL Great Ormond Street Institute of Child Health, London WC1N 1EH, UK; Dementia Research Centre, UCL Queen Square Institute of Neurology, London WC1N 3BG, UK; Department of Neurodegenerative Disease, UCL Queen Square Institute of Neurology, London WC1N 3BG, UK; UK Dementia Research Institute, University College London, London W1T 7NF, UK; Reta Lila Weston Institute, UCL Queen Square Institute of Neurology, London WC1N 1PJ, UK; Institute for Advanced Study, The Hong Kong University of Science and Technology, Hong Kong SAR, China; Department of Psychiatry and Neurochemistry, Institute of Neuroscience and Physiology, University of Gothenburg, Gothenburg 431 41, Sweden; Department of Medical Epidemiology and Biostatistics, Karolinska Institutet, Stockholm 171 77, Sweden; Department of Psychiatry and Neurochemistry, Institute of Neuroscience and Physiology, University of Gothenburg, Gothenburg 431 41, Sweden; Department of Neurodegenerative Disease, UCL Queen Square Institute of Neurology, London WC1N 3BG, UK; UK Dementia Research Institute, University College London, London W1T 7NF, UK; Clinical Neurochemistry Laboratory, Sahlgrenska University Hospital, Mölndal 413 45, Sweden; Hong Kong Center for Neurodegenerative Diseases, Clear Water Bay, Hong Kong SAR, China; Wisconsin Alzheimer’s Disease Research Center, University of Wisconsin School of Medicine and Public Health, University of Wisconsin-Madison, Madison, WI 53792, USA; Translational Mass Spectrometry Research Group, UCL Great Ormond Street Institute of Child Health, London WC1N 1EH, UK; Department of Clinical and Movement Neurosciences, UCL Queen Square Institute of Neurology, London NW3 2PF, UK

**Keywords:** apolipoprotein E, isoform, Alzheimer’s disease, ethanolamine plasmalogen, lipidomics

## Abstract

The ε4 allele of the *APOE* gene, encoding the E4 isoform of apolipoprotein E, is the leading genetic risk factor for late-onset Alzheimer’s disease. While many potential mechanisms have been proposed to explain this risk, no dominant or unifying process has yet emerged. Here, we explore the primary function of apolipoprotein E in lipid transport and metabolism, by examining its lipid association properties, to establish whether they show isoform dependence and thereby could mediate Alzheimer’s risk. We focus on ethanolamine plasmalogen, a phospholipid subclass known to be depleted in Alzheimer’s disease brain. We purified apolipoprotein E from human cerebrospinal fluid by immunoprecipitation using an anti-pan-apolipoprotein E monoclonal antibody bound to magnetic beads, then conducted lipidomic and proteomic analyses of the precipitates by mass spectrometry. The cerebrospinal fluid samples were obtained from cognitively intact, relatively young individuals with no evidence of amyloid pathology and with known apolipoprotein E isoform status (E3E3, *n* = 5; E3E4, *n* = 4; E4E4, *n* = 5). The molar ratio of ethanolamine plasmalogen to apolipoprotein E was 29.5% lower for E4E4 than for E3E3 (*P* = 0.007) with a biological gradient: E3E3 > E3E4 > E4E4 (*P* = 0.03). No similar trends and differences were found for phosphatidyl ethanolamine, a chemically related lipid (*P* = 0.5). Compared to E3E3, the molar ratio of ethanolamine plasmalogen to phosphatidyl ethanolamine was significantly reduced for E3E4 (*P* = 0.0016) and E4E4 (*P* = 0.0001). The latter deficiency was similar in magnitude to that found in Alzheimer’s disease brain relative to control. The finding that ethanolamine plasmalogen is depleted in apolipoprotein E4 relative to E3 strengthens the view that brain deficiency of this same lipid contributes to Alzheimer’s disease causation, rather than being an effect of the neurodegeneration. Simultaneously, these results supply a potential mechanism for the risk of E4 versus E3, the former being less able to counteract the tissue defect. The apolipoprotein E4 lipid depletion cannot itself be a consequence of Alzheimer’s disease, since cerebrospinal fluid samples were taken from individuals with no evidence of the condition. The biological gradient in ethanolamine plasmalogen deficiency mirrors the relationship of Alzheimer’s disease risk (odds ratio) to E4 allelic dose. Ethanolamine plasmalogen deficiency could be linked to, or indeed drive, several metabolic pathways implicated in Alzheimer’s pathogenesis, including amyloid-beta deposition and cholesterol dysregulation. Future studies should extend approaches to therapeutic intervention in Alzheimer’s disease which attempt to reverse this lipid abnormality.

## Teaser

Apolipoprotein E4 is the strongest genetic risk factor for late-onset Alzheimer’s disease. Here, Hällqvist *et al.* find an isoform-dependent gradient in the association of ethanolamine plasmalogen, a phospholipid implicated in Alzheimer’s pathogenesis, with apolipoprotein E (E3E3 > E3E4 > E4E4) and relate this lipid trend to these isoforms’ differential disease risk.

## Graphical Abstract

**Graphical Abstract:**
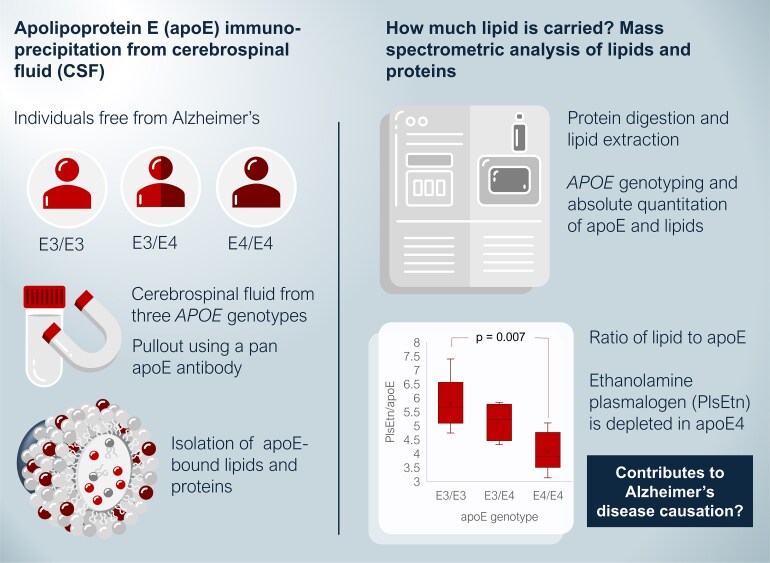

## Introduction

The ε4 allele of the apolipoprotein E (*APOE*) gene, encoding the E4 isoform of apoE protein, is the most significant genetic risk factor for late-onset Alzheimer’s disease,^1^ which is the most common cause of dementia worldwide.^2^ The presence of the *APOE* ε4 allele confers risk relative to the more prevalent ε3 (which encodes E3), whereas the rarer ε2 (encoding E2) is protective against the disease.^3^ Thus, if the Alzheimer’s odds ratio of apoE3E3 is 1, then that of apoE3E4 is typically 3–4 (with some variation dependent on study population), whereas that of apoE4E4 is usually about 12–15 (in white populations) and that of apoE2E3 ∼0.6.^4,5^

Human apoE is a 299-amino acid glycoprotein in which the differences between the E2, E3 and E4 isoforms relate to whether there is a cysteine or an arginine residue at positions 112 and 158. Hence, apoE2 is Cys112/Cys158, apoE3 is Cys112/Arg158 and apoE4 Arg112/Arg158.^6,7^ Despite some uncertainties, the secondary structure of all three isoforms is known to comprise a four-helix bundle in the N-terminal domain, a hinge region and a C-terminal domain, which is hydrophobic and thought to bind lipid. This structure is consistent with the primary physiological role of apoE in phospholipid and cholesterol transport and metabolism, both in the CNS and peripherally, although many other biological functions have been ascribed to it.^8^ Likewise, diverse apoE isoform-dependent metabolic responses have been implicated in Alzheimer’s disease pathogenesis. Indeed, the risk of apoE4 may be mediated by a combination of gain of toxic functions and loss of protective factors.^9^ Such mechanisms would include those which influence amyloid-beta (Aβ) deposition, the central pathway in our understanding of Alzheimer’s pathology,^10,11^ and others which seem amyloid-independent.^8^

While there may be more than one process underlying apoE4 risk, there is logic in attempting to identify a dominant or unifying mechanism, not least to aid therapeutic strategies. To this end, we here revisit the primary function of apoE in lipid biochemistry. An added impetus for doing so is provided by genome-wide association studies^12,13^ and more recent molecular genetic approaches,^14^ which have implicated other lipid-regulatory genes, particularly those encoding phospholipid transporters,^14^ as Alzheimer’s risk factors.^15^

**Figure 1:**
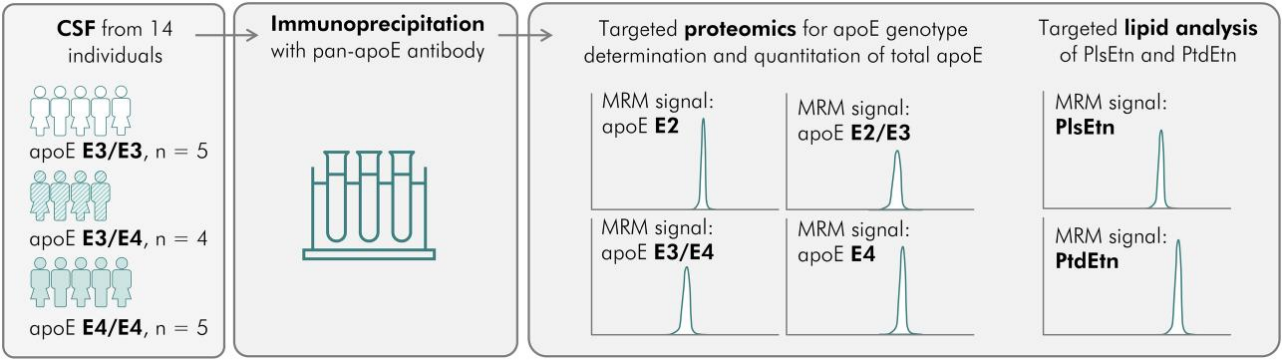
**Experimental workflow.** CSF samples from participants, classified by apolipoprotein E (apoE) isoform, underwent immunoprecipitation using anti-pan-apoE monoclonal antibodies. The precipitates were then analysed for the phospholipid class of ethanolamine glycerophospholipids (ethanolamine plasmalogen subclass, PlsEtn and phosphatidyl ethanolamine subclass, PtdEtn) and for apoE by mass spectrometry using multiple reaction monitoring (MRM) (see ‘Materials and methods’ section).

Our specific goal was to examine whether apoE isoforms differ in their phospholipid association properties. To achieve this aim, we devised a methodology for purifying apoE from human CSF by immunoprecipitation (IP), using an anti-pan-apoE monoclonal antibody bound to magnetic beads, and then conducting lipidomic and proteomic analyses of the precipitate by mass spectrometry. Our workflow is summarized in Fig. 1. We targeted the phospholipid subclass of ethanolamine plasmalogens (PlsEtn) in these experiments, because PlsEtn deficiency has been detected previously in Alzheimer’s disease brain.^16-19^ As a form of internal control, we also analysed phosphatidyl ethanolamines (PtdEtn), the other major subclass of ethanolamine glycerophospholipids.

## Materials and methods

### Participants and samples

CSF samples were retrieved from the St. Göran bipolar project, a longitudinal study of bipolar syndromes, described in detail elsewhere.^20-22^ In brief, the study enrols adult patients with bipolar spectrum disorder from outpatient clinics and healthy control individuals randomly selected from the population. Lumbar puncture took place between 0900 and 1000 h, after an overnight fast, by inserting a spinal needle into the L3/L4 or L4/L5 interspace in the seated position. The sample tube was inverted to avoid concentration gradients, aliquoted and stored at −80°C pending analysis. No sample was subjected to freeze-thaw cycles. Patients were in a mood-stabilized phase at the time of sampling and remained on current medication. All study participants provided oral and written consent. The study was conducted in accordance with the Declaration of Helsinki and approved by the Stockholm Regional Ethics Committee. *APOE* genotypes were obtained from two single-nucleotide polymorphisms (rs7412 and rs429358) as described previously.^23^ For this study, CSF samples stratified by *APOE* alleles were randomly selected [*n* = 5 ε3ε3, *n* = 5 ε3ε4 (but see below), *n* = 5 ε4ε4]. The demographic characteristics of these genotype groups are summarized in Table 1.

**Table 1:** Demographics of participants providing analysed samples

	E3/E3	E3/E4	E4/E4	ANOVA *P*
*n*	5	4	5	
Aβ42 (pg/mL)	274.6 ± 35.0	255.0 ± 50.1	232.8 ± 41.0	0.32
Age (years)	41.2 ± 13.8	37.0 ± 8.8	40.8 ± 16.9	0.89
BMI	22.3 ± 3.6	24.7 ± 2.6	22.3 ± 3.7	0.51
Sex	2 M/3F	2 M/2F	2 M/3F	
Diagnostic group	3BPD/2Ctrl	3BPD/1Ctrl	2BPD/3Ctrl	

Means ± standard deviations are reported. Single factor ANOVA was used to evaluate differences between the *APOE* genotype groups.

Aβ42, amyloid-beta 42; BMI, body mass index; M, male; F, female; BPD, bipolar disorder; Ctrl, controls.

*A priori* statistical power calculations, with the aim of judging appropriate genotype/isoform group size, were based on the null hypothesis that the difference in PlsEtn/PtdEtn (expressed as molar ratios—see below) for apoE4E4 relative to apoE3E3 would be of comparable magnitude to that seen for Alzheimer’s disease brain (temporal cortex) relative to control. Effect sizes, means and standard deviations were therefore sourced directly from these previously published brain tissue values,^16^ yielding a target range of *n* = 2–6 for each genotype/isoform group (α = 0.05; statistical power to be achieved = 0.8). The actual group size (*n* = 5) fell towards the upper end of this range and power was further strengthened by the addition of a potential intermediate group (apoE3E4) to detect trends (see ‘Discussion’ section). Other considerations in the setting of group size included striking a balance between the need for sufficient power to test this proof-of-concept study and the use of relatively scarce and valuable CSF sample resources, obtained by invasive means, diverted from other studies and required in significant volumes in a labour-intensive analysis. Finally, there was the recognition that some CSF samples, albeit of lesser provenance, were to be used, indeed sacrificed, in the methodological development phase of our work.

### Apolipoprotein E immunoprecipitation

#### Coupling of anti-apoE antibodies to Dynabeads

After removal of potential antibody aggregates by centrifugation, anti-pan-apoE monoclonal antibodies (Abcam, ab227993) were covalently bound to Dynabeads M-270 Epoxy with the Dynabeads Antibody Coupling Kit (Thermo Fisher Scientific, 14311D) at a ratio of 5 μg of IgG per mg of beads in 2-mL Eppendorf Safe-Lock Tubes (0030120094) as recommended by the manufacturer. After coupling on a tube rotator at 37°C overnight, the beads were collected on a magnet, the supernatant was removed and the beads were successively washed with HB, LB and SB buffer from the kit (900 μL of buffer per 20 mg of beads) by gentle vortexing. The short wash with SB buffer was repeated once, followed by a longer wash with SB buffer on a tube rotator for 15 min. Next, the beads were collected on a magnet, the supernatant was removed and the beads were washed with phosphate-buffered saline (PBS) six times by gentle vortexing. After the last collection of the beads on a magnet and removal of the supernatant, the anti-apoE-coupled Dynabeads were resuspended in PBS at a concentration of 1 mg of beads per 100 μL of PBS.

#### Immunoprecipitation of apoE and associated lipids

CSF samples, stored at −80°C, were thawed protected from light and potential aggregates were removed by centrifugation at 4°C. ApoE and associated lipids/proteins in 400 μL of CSF were immunoprecipitated with 500 μL of anti-apoE-coupled Dynabead suspension in 1.5-mL reaction tubes (Greiner Bio-one; 616201) on a tube rotator at room temperature for 1 h protected from light. After immunocapture, the beads were collected on a magnet, the supernatant was removed and the beads were washed six times with 900 μL PBS by gentle vortexing. After the final collection of the beads on a magnet and removal of the supernatant, the beads were briefly centrifuged, and all traces of supernatant were removed. The beads with immunoprecipitated apoE and associated lipids were stored at −80°C and analysed during the same working week. A step-by-step protocol of the apoE IP has been published elsewhere.^24^

### Preparation of lipids and proteins for mass spectrometry

#### Extraction of apoE-bound lipids

ApoE-bound lipids, isolated by IP, were prepared for mass spectrometric analysis by single-phase extraction using methanol^25^ containing the heavy isotope-labelled internal standard PtdEtn (C15:0/18:1)-d7 (Avanti Polar lipids, 791638), as described in detail in the Supplementary materials and methods. Two separate calibration curves, each ranging from 0 to 10 ng/μL, were created from a PtdEtn mixture (Matreya, 1069) and from PlsEtn (C18:0/18:1) (Avanti Polar lipids, 85275P).

#### Digestion and solid phase extraction of peptides for proteomic analyses

Enzymatic digestion of the protein bound to the magnetic beads and subsequent solid phase extraction of peptides are described in the Supplementary materials and methods. A calibration curve was created for targeted apoE assay from digested, synthetic peptide standards for the apoE peptides LAVYQAGAR, LGADMEDVR, CLAVYQAGAR, LGADMEDVCGR and AATVGSLAGQPLQER, all from GenScript. The range of the calibration curve was 0–0.1 µM.

### Instrumental analysis

#### Mass spectrometric analysis of lipids

Lipid analyses were conducted in accordance with guidelines and standards published by the International Lipidomics Society^26^ (see Supplementary material—International Lipidomics Society checklist). Samples were analysed using a Waters ACQUITY Liquid Chromatography Quaternary Solvent Manager system coupled to a Waters Xevo TQ-S mass spectrometer. Two microlitres of sample was injected and analytes were separated on a Waters ACQUITY BEH HILIC 1.7 µm column, 2.1 × 50 mm, equipped with a VanGuard column of the same chemistry, operating at 45°C. For other conditions and settings, including solvent systems and elution profile, see Supplementary materials and methods.

PlsEtn and PtdEtn species were detected using multiple reaction monitoring in positive electrospray ionization (ESI) mode. The carbon chain length and distribution of double bonds on the alkenyl and acyl chains of the PlsEtn molecular species were identified by monitoring two transitions per compound, for the *sn*-1 alkenyl chain following the reaction from the protonated precursor ion to the product ion of the *sn*-1 ether and the phosphoethanolamine headgroup and for the *sn*-2 acyl chain following the reaction from the protonated precursor ion to the fragment corresponding to the *sn*-2 acyl chain ester bound to prop-2-en-1-ol.^27^ PtdEtn species were identified by the reaction from the protonated precursor ion to the product ion resulting from neutral loss of the phosphoethanolamine headgroup. Supplementary Tables S1 and S2 show the monitored transitions.

#### Targeted apoE analysis by mass spectrometry

Samples were analysed using a Waters ACQUITY Liquid Chromatography Binary Solvent Manager system coupled to a Waters Xevo TQ-S mass spectrometer. Five microlitres of sample was injected and peptides were separated on a Waters CORTECS UPLC® C18 + 1.6 µm column, 2.1 × 50 mm, equipped with a VanGuard column of the same chemistry, operating at 45°C. See Supplementary materials and methods for further details of conditions and settings.

To determine and quantify apoE isoforms, four different peptides, corresponding to genotype-specific alterations in the apoE protein’s amino acid sequence for E2, E3 and E4, were monitored alongside one peptide unaffected by the apoE genotypes. The peptides were measured by multiple reaction monitoring in positive ESI mode, using in-house assays as described previously.^28^ Each peptide was monitored by two transitions as shown in Supplementary Table S3.

Our targeted apoE proteomics assay allowed identification of apoE isoforms as well as their quantitation, and therefore, our methodology had a built-in internal check of *APOE* genotype. All proteomic (and lipidomic) analyses were conducted blinded for *APOE* genotype, as determined by conventional means (see ‘Participants and samples’ section). Results were included only if there was 1:1 correspondence between apoE isoforms identified by proteomics and *APOE* genotype determined by conventional molecular genetics, which was the case for all but one sample, hence ultimately *n* = 14 not 15 (ε3ε3: *n* = 5, ε3ε4: *n* = 4, ε4ε4: *n* = 5). The exception was a sample originally labelled as ε3ε4 based on molecular genetics but identified as E3E3 by proteomics. The inclusion or exclusion of this one anomalous sample did not affect our general conclusions.

#### Untargeted proteomics mass spectrometry

Peptides were separated using a Waters nanoACQUITY liquid chromatography system equipped with a 180 µm × 20 mm, 5 µm Symmetry C18 trap column (Waters) coupled to a 75 µm × 150 mm, 1.7 µm Peptide BEH C18 column (Waters). The column temperature was 45°C. Other conditions and settings are given in the Supplementary materials and methods. The eluted peptides were detected on a Synapt-G2-Si (Waters) fitted with a nano-electrospray ion source. Data were acquired in positive MS^E^ mode within the m/z range 50–2000, from 0 to 60 min. In the low energy acquisition, a fixed collision voltage of 4 V was applied with a 1 s scan time. The high energy acquisition applied a collision energy ramp from 15 to 40 V, with a scan time of 1 s. The lock mass utilized for mass correction consisted of [glu1]-fibrinopeptide B, which was infused at a concentration of 500 fmol/µL and a flow rate of 0.3 µL/min. The doubly charged precursor ion, m/z 785.8426, was acquired every 30 s.

### Data processing

#### Analysis of targeted mass spectrometric data

Data were analysed using TargetLynx (Waters) or an in-house application written in the programming language Python. Peak areas were exported to Excel and the ratios between analytes and internal standards were calculated prior to concentration determination, relating the analyte to internal standard ratio to the calibration curves using the linear equation *y* = *ax*  *+*  *b*, where *y* is analyte to internal standard ratio, *a* is the slope, *x* is the concentration and *b* is the intercept. The concentrations of lipids and peptides were expressed in molar terms to allow for comparison of the different compounds.

#### Analysis of untargeted mass spectrometric data

Data were processed using Progenesis QI for proteomics (Waters). The Ion Accounting workflow was applied, with the database used for identifications being UniProt’s Canonical Human Proteome (exported 2022). The settings were as follows: the digestion enzyme was trypsin, carbamidomethyl on cysteines was a fixed modification, variable modifications included deamidation of glutamine and asparagine and oxidation of tryptophan and pyrrolidone carboxylic acid on the N-terminus. A false discovery rate of 4% or less was accepted, and the identification tolerance was restricted to at least two fragments per peptide, three fragments per protein and one peptide per protein.

#### Statistical analysis

Statistical analyses were performed in GraphPad Prism (version 6.0.1, GraphPad Software, San Diego, California USA, www.graphpad.com). The demographics of the sample groups were evaluated by single factor ANOVA. To estimate the number of lipid molecules associated with apoE for the different isoforms E3E3, E3E4 and E4E4, molar ratios between each lipid species and total apoE were calculated. The molar ratios were evaluated for normal distribution applying D'Agostino’s normality test. Differences between isoforms were assessed using one factor ANOVA followed by Fisher’s least significant difference test. The relationship between lipid/apoE molar ratio and E4 allelic dose was investigated using product moment correlation coefficients. The Benjamini–Hochberg procedure was applied to account for multiple testing with 5% false discovery rate.

## Results

### Genotype group characteristics

To avoid any potential influence of neurodegenerative disease on the lipidomics or proteomics, we analysed CSF samples from participants with normal cognitive function. Thus, on the WAIS (Wechsler Adult Intelligence Scale—version III), all but one individual scored ≥90 for full-scale intelligence quotient (mean 112.2, SD 16). For working memory index, all but the same individual scored ≥86 (mean 102.5, SD 15.8). There was no significant difference between genotype groups (ε3ε3, *n* = 5; ε3ε4, *n* = 4; ε4ε4, *n* = 5) for age, sex or health status, including CSF Aβ42 levels (Table 1).

In preliminary experiments, we also quantified CSF apoE concentrations and did detect an isoform-dependent gradient: [E2] > [E3] > [E4] (Supplementary Fig. S1: the data in this figure include results obtained from the samples which are the focus of the present study, combined with those from samples analysed in earlier developmental stages of our methodology, some with ε2 genotypes).

### Lipidomic analysis of the immunoprecipitates

**Figure 2:**
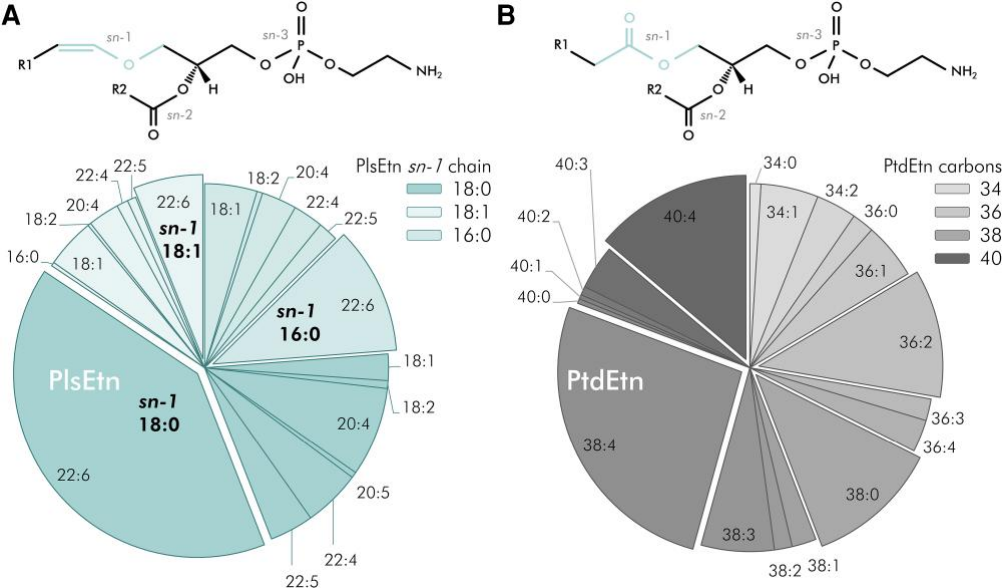
**Ethanolamine glycerophospholipid species associated with apolipoprotein E.** (**A** and **B**) Pie charts of the proportions (expressed as mole % of total in subclass) of different species of the ethanolamine plasmalogen (PlsEtn) and phosphatidyl ethanolamine (PtdEtn) subclasses, respectively, associated with apolipoprotein E. Insets show the generic molecular structures of PlsEtn and PtdEtn (see text for details of structural differences). The proportions of the different lipid species were derived from the entire sample set. For PlsEtn (**A**), the shading of the groups of sectors in the pie chart varies according to *sn*-1 side chain (R1, with darker teal for 18:0, intermediate for 16:0 and paler for 18:1). The individual sectors are then labelled for *sn*-2 side chain (R2). For PtdEtn (**B**), the shading of the sectors reflects the lengths of the side chains, with darker shading for a higher total number of carbon atoms.

Each apoE-IP underwent lipidomic analysis for ethanolamine glycerophospholipids—both PlsEtn and PtdEtn—and quantitative proteomic analysis for apoE (Fig. 1 and see ‘Materials and methods’ section). The proportions of different species of PlsEtn and PtdEtn associated with apoE are shown in Fig. 2, along with the generic molecular structures of these two phospholipid subclasses. To summarize the structural differences, for both PtdEtn and PlsEtn, there is a phosphoethanolamine headgroup at the *sn*-3 position of the glycerol backbone of the lipid, with a fatty acyl group at *sn*-2. For PtdEtn, there is another fatty acyl group at *sn*-1, whereas for PlsEtn, the *sn*-1 position is occupied by a fatty alcohol group, linked by a vinyl ether bond (hence an alkenyl rather than acyl chain). The molecular species of PlsEtn vary in their side chains at the *sn*-1 and *sn*-2 positions. Generally, the *sn*-1 position is occupied by saturated (C16:0, C18:0) or monounsaturated (C18:1) chains, whereas those at *sn*-2 are more diverse and more likely to be polyunsaturated.

Of the 40 monitored molecular species of PlsEtn (Supplementary Table S1) at least 20 were associated with the apoE-IP, but the C18:0/22:6 form was dominant, accounting on average for 40.3 mole % of the total PlsEtn (Fig. 2A). For PtdEtn, at least 18 of the 28 monitored species (Supplementary Table S2) were identified in the apoE-IP, with less clear-cut species dominance, though the C38:4 form accounted for >26 mole % of the total PtdEtn (Fig. 2B).

Lipid yields in control IP experiments using magnetic beads without anti-apoE antibody attached were <7% of those obtained with antibody present. Put another way, the presence of >93% of the ethanolamine glycerophospholipid in the precipitate was apoE-dependent.

### Phospholipid-apolipoprotein E molar ratios

**Figure 3:**
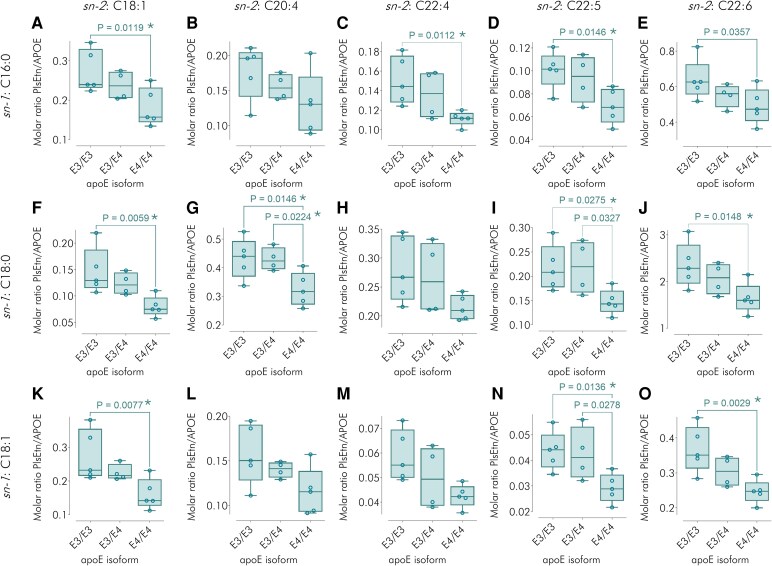
**Ethanolamine plasmalogen-apolipoprotein E molar ratios.** (**A–O**) Molar ratios of ethanolamine plasmalogen (PlsEtn) to apolipoprotein E (apoE), according to apoE isoform (E3E3 *n* = 5, E3E4 *n* = 4 or E4E4 *n* = 5) and to PlsEtn molecular species. Box-and-whisker plots where the whiskers show the minimum and maximum values and the boxes show the 25th percentile, the median and the 75th percentile. Each data point (small open circle) represents a CSF sample from an individual participant. Nominal *P*-values comparing PlsEtn/apoE molar ratios for the three different isoforms were calculated using single factor ANOVA followed by Fisher’s least significant difference test and are shown in the plots, annotated by an asterisk for the comparisons which were significant post multiple testing correction using the Benjamini–Hochberg procedure with false discovery rate set to 5% (see ‘Materials and methods’ section).

There was a significant reduction (*P* < 0.05) in PlsEtn/apoE molar ratio comparing apoE4E4 with apoE3E3 in 10/20 molecular species of PlsEtn, post multiple testing correction (Fig. 3), including the dominant C18:0/22:6 form (Fig. 3J). For PtdEtn/apoE molar ratios, no similar trends were observed. Indeed, for 6 of the 18 identified PtdEtn species PtdEtn/apoE4E4 exceeded PtdEtn/apoE3E3. Only 3/18 PtdEtn species showed a significant reduction (*P* < 0.05) in PtdEtn content comparing apoE4E4 with apoE3E3, and these did not include the most abundant species (C38:4) (Supplementary Fig. S2).

**Figure 4:**
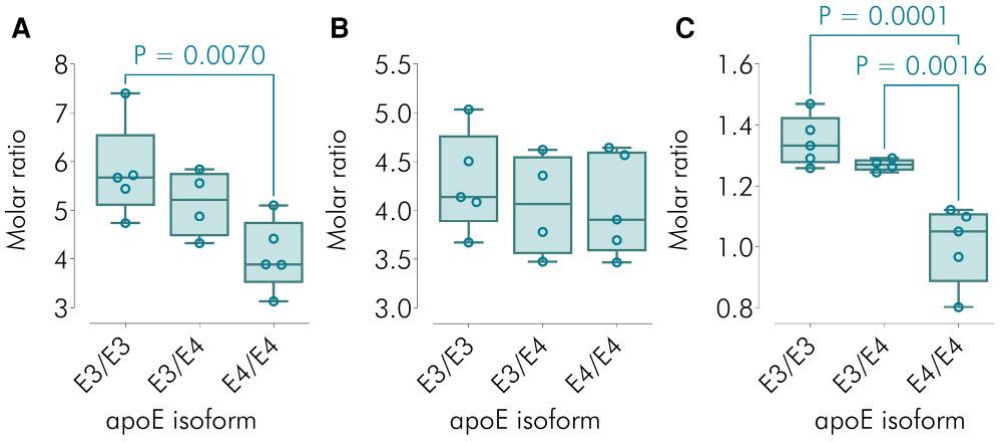
**Molar ratios of ethanolamine plasmalogen (PlsEtn) and phosphatidyl ethanolamine (PtdEtn) to apolipoprotein E (apoE) and to each other.** (**A** and **B**) Molar ratios of PlsEtn/apoE and PtdEtn/apoE, respectively, according to apoE isoform (E3E3 *n* = 5, E3E4 *n* = 4 or E4E4 *n* = 5). (**C**) Molar ratios of PlsEtn/PtdEtn, according to apoE isoform. All lipid/protein and lipid/lipid ratios are for the total lipid (sum of all detected species) in the relevant subclass. Box-and-whisker plots where the whiskers show the minimum and maximum values and the boxes show the 25th percentile, the median and the 75th percentile. Each data point (small open circle) represents a CSF sample from an individual participant. Nominal *P*-values were calculated using single factor ANOVA followed by Fisher’s least significant difference test and are shown in the plots.

Combining all identified molecular species of PlsEtn, there was a 29.5% reduction in PlsEtn/apoE molar ratio for apoE4E4 compared to apoE3E3 (*P* = 0.007) (Fig. 4A). When a similar overall total was calculated for PtdEtn, there was no significant isoform dependence of PtdEtn/apoE molar ratio (*P* = 0.5) (Fig. 4B). The difference between PlsEtn and PtdEtn was further underlined by calculating PlsEtn/PtdEtn molar ratios, for which (apo)E3E4 > E4E4 and E3E3 > E4E4 (*P* << 0.01 in both cases) (Fig. 4C).

Comparison of PlsEtn/apoE4E4 molar ratios with PlsEtn/apoE3E4 and PlsEtn/apoE3E3 showed a biological gradient in PlsEtn content with apoE isoform of E3E3 > E3E4 > E4E4 for 17 of the 20 identified molecular species of PlsEtn (Fig. 3). For 13 PlsEtn molecular species and for total PlsEtn, there was a significant negative correlation between lipid/apoE molar ratio and E4 allelic dose (0, 1 or 2) (where *r*, the product moment correlation coefficient, varied between −0.75 and −0.58; Table 2). In contrast, only three PtdEtn species showed a similar negative correlation. Among the PlsEtn molecular species, a significant negative correlation was predominantly found for those with polyunsaturated *sn*-2 moieties, C22:4, C22:5 and C22:6.

**Table 2:** Significant correlations between apoE4 allelic dose and PlsEtn/apoE molar ratio

PlsEtn molecular species	Correlation *P*-value	Product moment correlation coefficient
Total	0.031	−0.686
C16:0/18:1	0.031	−0.669
C16:0/22:4	0.031	−0.673
C16:0/22:5	0.032	−0.647
C16:0/22:6	0.046	−0.584
C18:0/18:1	0.031	−0.712
C18:0/20:4	0.033	−0.624
C18:0/22:5	0.047	−0.576
C18:0/22:6	0.032	−0.655
C18:1/18:1	0.031	−0.700
C18:1/20:4	0.039	−0.603
C18:1/22:4	0.033	−0.630
C18:1/22:5	0.033	−0.636
C18:1/22:6	0.031	−0.753

Product moment correlation coefficients were calculated. The *P*-values were adjusted for multiple testing applying the Benjamini–Hochberg procedure with alpha = 0.05. Values are given for all ethanolamine plasmalogen (PlsEtn) molecular species where the correlation coefficient between apoE4 (apolipoprotein E4) allelic dose and PlsEtn/apoE molar ratio achieved statistical significance (defined as *P* < 0.05) and for total PlsEtn.

### Untargeted proteomic analysis of the immunoprecipitates

**Figure 5:**
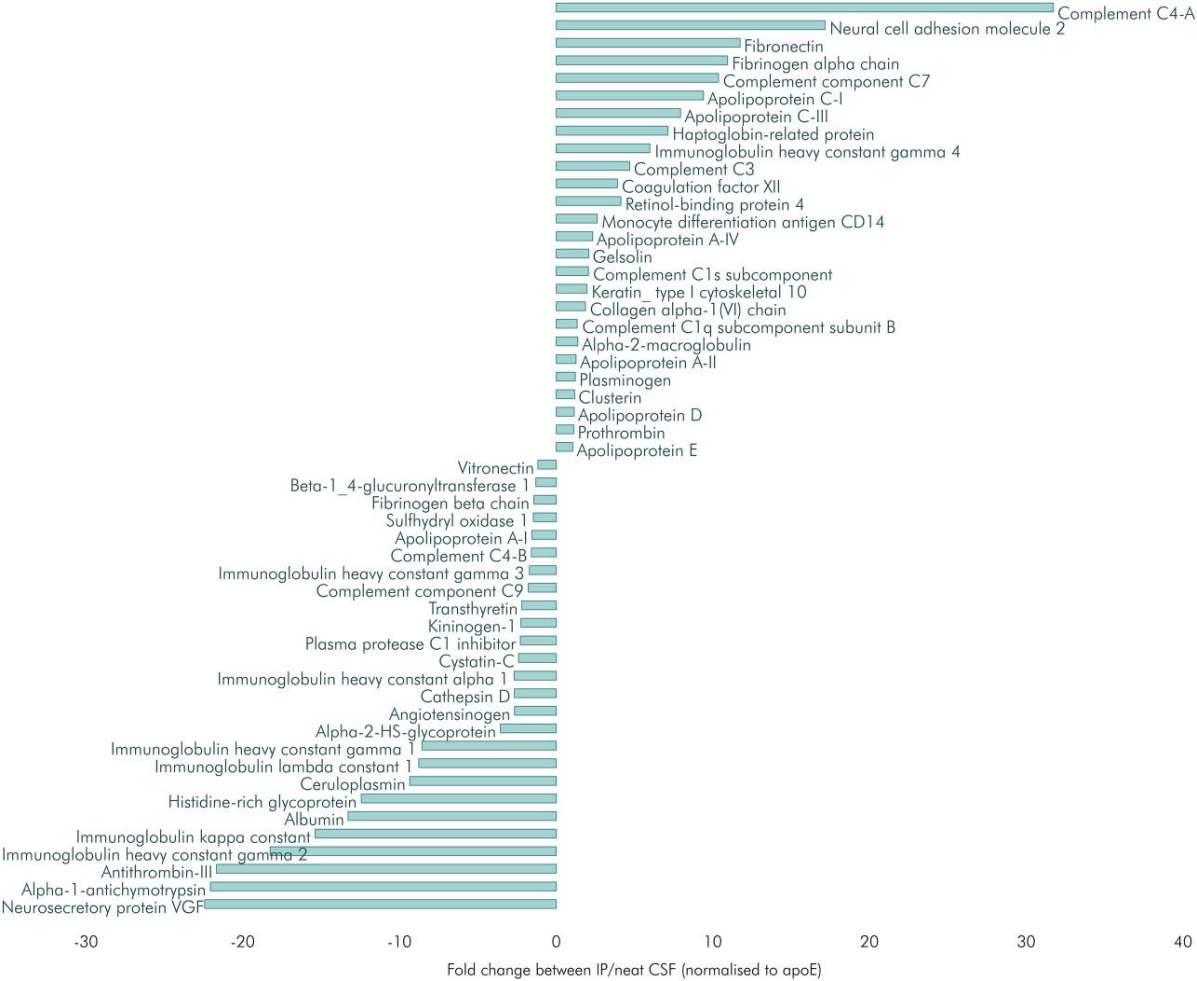
**Relative enrichment in apolipoprotein E (apoE) immunoprecipitation (IP) pulldown.** Protein levels detected by untargeted mass spectrometry in neat CSF (*n* = 14) were compared to paired IP CSF (*n* = 14). The protein levels have been normalized to each sample’s apoE concentration, and the graph shows the ratio of proteins in IP to neat CSF. No statistical tests were applied to these ratios; the figure being intended purely as an illustrative comparison. Positive values indicate that a protein was enriched by the IP, while negative values represent proteins which were pulled down by IP but are present at lower levels compared to apoE than in neat CSF.

We assessed the relative degree of apoE enrichment and purification achieved by the IP protocol, using untargeted mass spectrometry (Fig. 5 and see ‘Materials and methods’ section), comparing neat CSF with paired IP CSF. Although there was an approximately 15-fold enrichment of apoE relative to albumin, the most abundant CSF protein (from apoE/albumin = 13% in neat CSF to 191% post-IP), other proteins were also enriched in the precipitate. These included several potentially lipid-bearing proteins, notably apolipoproteins A-II, A-IV, C-I, C-III, D and J (clusterin) (Fig. 5). Amyloid precursor protein (APP) was present in neat CSF but was not found in IP CSF by our proteomic analysis (although the methodology was not geared to detect peptides from the Aβ42 amino acid sequence of the precursor protein).

## Discussion

In this study, we explored the relationship between apoE isoform and this lipoprotein’s lipid association properties. We found isoform dependence of binding for PlsEtn, a specific phospholipid subclass, with apoE4 PlsEtn levels being significantly lower than those associated with apoE3. Before considering whether apoE4 PlsEtn deficiency, relative to E3, may be involved in mediating Alzheimer’s disease risk, we examine first our rationale for selecting CSF as source of apoE for our experiments and PlsEtn as target lipid for analysis.

Previous attempts to characterize and quantify lipids associated with apoE used lipoprotein purified from primary cultures of mouse astrocytes derived from transgenic mice expressing human apoE isoforms.^29,30^ The decision to obtain apoE directly from human extracellular fluid, instead of murine cell culture medium, depended on our development of an immunoprecipitation/mass spectrometric technique sufficiently sensitive to measure individual lipid and protein species at sub-picomolar levels in small sample volumes, but had at least two inherent advantages. First, there would be an increased likelihood of preserving lipid–protein and protein–protein interactions with apoE relevant to human physiology. Second, any isoform-dependent biological gradient in lipid association properties would be better evidenced in a system with three options (E3E3 versus E3E4 versus E4E4) as opposed to just two for transgenic mice (E3 versus E4).

Apart from the obvious proximity to brain tissue, we chose to examine apoE in human CSF rather than plasma because it is the predominant apolipoprotein in the former extracellular fluid, whereas it is a relatively minor constituent of the latter.^31^ There is also evidence that CNS and systemic apoE form distinct metabolic pools, the apolipoprotein being unable to cross the blood–brain barrier.^32^ Furthermore, apoE tends to associate with different-sized lipoprotein particles in blood and CSF.^33^ These association properties, including with other proteins, could influence overall lipoprotein lipid composition.

It could be of interest to investigate the lipids associated with plasma apoE, given the finding of systemic alterations in PlsEtn^34,35^ and other lipids^36^ in Alzheimer’s disease. But such a study would likely require a more complex apoE purification procedure, which could disrupt physiological relationships between lipid-bearing proteins and hence produce misleading results, and might become more of an exercise in biomarker discovery than an examination of Alzheimer’s disease pathogenesis. That said, a study correlating lipid species in whole (unpurified) plasma with *APOE* genotype found an ε2 > ε3 > ε4 gradient in concentration for plasmalogens and allied ether lipids.^37^

Regarding choice of lipid as target for analysis, both phospholipids and cholesterol are associated with apoE, but we focused on the former, and particularly PlsEtn, for several reasons. First, other lipid-binding proteins which have been implicated as Alzheimer’s risk factors are more specifically phospholipid transporters.^12-14^ Second, although there has been recent literature on apoE isoform-driven variations in cholesterol metabolism in oligodendrocytes,^38^ astrocytes^39,40^ and microglia^40^ in Alzheimer’s pathogenesis, such metabolic processes may themselves depend on PlsEtn levels (see below). Third, PlsEtn is a major constituent of brain lipids, accounting for >30 mole % of the total phospholipid in normal human white matter (>70 mole % of the ethanolamine glycerophospholipids) and ∼20 mole % of the total phospholipid in grey matter.^41^ Much of this phospholipid is located in neuronal and glial cellular membranes and membrane instability has been implicated in Alzheimer’s pathogenesis (see below). While membrane bilayer stability is highly sensitive to PlsEtn mole fraction,^42^ it is relatively resistant to changes in cholesterol content.^43^ The final and most important reason for targeting PlsEtn in our analysis is the previous finding that this phospholipid is deficient in Alzheimer’s disease brain.^16-19^

### Brain PlsEtn deficiency in Alzheimer’s disease—cause or effect?

Since the original descriptions of Alzheimer’s brain PlsEtn deficiency,^16,17^ there have been divergent views about whether this metabolic defect contributes to disease causation or is merely a downstream consequence of the condition.^44^ The observation in the present study of a parallel lack of PlsEtn associated with apoE4E4, relative to apoE3E3, analysing samples from individuals who *do not* have the disease, lends weight to the former view. Thus, in broad terms, one possible mechanism for the increased Alzheimer’s risk of E4E4 is that this isoform is less able to counteract or restore brain PlsEtn deficiency. Although ultimately it may be of value to investigate apoE lipidomics in Alzheimer’s patients, we avoided such samples in the present study precisely because of the possible influence of disease progression on their lipid profiles.

Despite relatively small genotype/isoform group sizes, we found significant differences between apoE3E3 and apoE4E4 for PlsEtn/apoE molar ratio (Fig. 4A). No such differences were seen for PtdEtn (Fig. 4B), another ethanolamine glycerophospholipid, with a superficial structural resemblance to PlsEtn, but distinct biosynthesis and degradation,^44^ thereby providing evidence of lipid specificity of the PlsEtn findings. Furthermore, for the majority of detected PlsEtn molecular species, there was an inverse link between their degree of association with apoE and E4 allelic dose (Fig. 3). This relationship appears linear (Fig. 4A and Table 2), suggesting a log-linear connection between Alzheimer’s disease risk, expressed as odds ratios, and PlsEtn/apoE, totalling all PlsEtn molecular species (see Supplementary discussion for derivation). Finally, comparing PlsEtn/PtdEtn molar ratio for apoE4E4 to that for apoE3E3 (Fig. 4C) yields a similar change in magnitude to that obtained comparing Alzheimer’s to control brain^16^ [(1.02 *cf*. 1.35) versus (0.87 *cf.* 1.26), respectively].

Our PlsEtn/apoE results cannot be dismissed simply as being consequences of CSF apoE concentration, because we quantified these concentrations and detected an isoform-dependent gradient: [E2] > [E3] > [E4] (Supplementary Fig. S1). This trend would serve only to accentuate the PlsEtn/apoE molar ratio findings. Previous studies have not uniformly confirmed such a gradient and, in some larger datasets, CSF apoE level was found not to vary according to *APOE* genotype. However, this earlier work was performed mainly in older individuals than those in the current analysis.^45-47^ Either way, the PlsEtn/apoE observations cannot be regarded merely as ‘compensating’ for differences in CSF apoE concentration.

Although our IP methodology permitted lipid-apoE association to be expressed in molar ratio terms, such results cannot be translated into absolute statements about the stoichiometry of the lipid–protein interaction. This is primarily because the IP protocol represents only a partial purification of apoE. Other proteins were present in the precipitate (Fig. 5), some of which could also be associated with lipid. Thus, apolipoproteins A-II, A-IV, C-I, C-III, D and J (clusterin) were enriched in the IP relative to apoE. Conversely, apoE was enriched relative to apolipoprotein A-I. Apolipoprotein B was not found in the precipitate yet is present in CSF (albeit in small quantities).^33^ The identification of apolipoproteins apart from apoE, and other proteins, in the IP is consistent with the view that apoE in CSF is a major scaffold protein, co-located in specific particles with some proteins such as clusterin, but also associating with multiple lipoprotein subpopulations, each a supramolecular array of lipid and protein species.^33^ Of course, finding proteins other than apoE in the precipitate could also be in part a consequence of non-specific protein binding to the magnetic beads used in the IP protocol. But the same cannot be said of the lipid associated with the apoE-IP, because control experiments using magnetic beads without anti-apoE antibody showed that the presence of >93% of the ethanolamine glycerophospholipid in the precipitate was apoE-dependent, either directly by binding to apoE or indirectly via associated proteins.

### Pathomechanisms

There are several potential mechanisms whereby brain PlsEtn deficiency could be implicated in Alzheimer’s disease causation. These include a direct effect of the lipid defect on the stability of neuronal and glial membrane bilayers,^42,48^ thereby contributing to neurodegeneration either independently or synergistically with amyloidogenesis. In the latter scenario, apoE4’s relative PlsEtn deficiency could mediate its increased Alzheimer’s risk by failing to ‘correct’ the lack of this lipid in CNS cell membranes, leading to destabilization of APP’s membrane milieu, rendering the precursor protein more accessible to enzymatic cleavage to Aβ.^48,49^ A second potential mechanism would be through diminished protection against reactive oxygen species, given the antioxidant properties of PlsEtn^50^ and the suggested role of oxidative stress in Alzheimer’s disease causation.^51,52^ In relation to our present results, it is noteworthy that apoE4 has reduced antioxidant activity compared to apoE3.^53^ Finally, the recent findings of apoE4-driven cholesterol dysregulation in oligodendrocytes,^38^ astrocytes^39,40^ and microglia^40^ suggest a further possible link with PlsEtn deficiency: apoE4 influences cellular cholesterol transport^39^ and so does PlsEtn deficiency.^54-56^

### Conclusion and future directions of study

We observe here that CSF PlsEtn-apoE association is apoE isoform-dependent, with a significant reduction in PlsEtn content comparing E4E4 with E3E3 and an E3E3 > E3E4 > E4E4 biological gradient. The magnitude of the PlsEtn deficiency of E4E4 relative to E3E3 is commensurate with that reported previously for Alzheimer’s disease post-mortem brain samples compared to controls,^16^ and there is evidence of lipid specificity as no similar defect is found for PtdEtn, another ethanolamine glycerophospholipid. Plausible mechanisms are advanced whereby PlsEtn deficiency could mediate the Alzheimer’s disease risk of apoE4, and our approach eliminates the possibility that this lipid defect is merely a consequence of the disease.

Future studies, as well as attempting to validate these findings in larger data groups, with a broader lipidomic focus, should include rarer apoE isoforms, containing E2, to determine whether PlsEtn-apoE2 association is enhanced relative to E3, in line with the lower Alzheimer’s risk of E2. The utility of the semilogarithmic relationship between Alzheimer’s odds ratio and PlsEtn/apoE (see above and Supplementary discussion) would be tested if the model holds even with isoforms not consisting solely of E3 and/or E4. The underlying biochemical basis of the PlsEtn deficiency also warrants further investigation, as does its relationship to other lipids with emerging implications in Alzheimer’s disease pathogenesis.^57^ Finally, nascent attempts to modulate brain PlsEtn levels therapeutically should be pursued.^18,44,58-60^

## Supplementary Material

fcag040_Supplementary_Data

## Data Availability

The mass spectrometry data that support the findings of this study have been deposited to the ProteomeXchange Consortium via the PRIDE partner repository with the dataset identifier PXD068681 and via the Panorama repository https://panoramaweb.org/APOE_IP_CSF.url. The in-house application used for data integration (‘mrmIntegrate’) was written in Python (version 3.8) and is publicly available to download via the GitHub repository https://github.com/jchallqvist/mrmIntegrate.
