# Supplementary material for: Plasmalogen deficiency and the Alzheimer’s disease risk of apolipoprotein E4
Source: Brain Commun. 2026 Feb 12;8(1):fcag040. doi: 10.1093/braincomms/fcag040 (PMC12946155; doi:10.1093/braincomms/fcag040)
Supplement: fcag040_Supplementary_Data [file fcag040_supplementary_data.pdf]

Plasmalogen deficiency and the Alzheimer's disease risk of apolipoprotein E4

Supplementary material

Contents:

Supplementary materials and methods	page 1
Supplementary discussion	page 3
Supplementary references	page 4
Supplementary Table 1	page 6
Supplementary Table 2	page 7
Supplementary Table 3	page 8
Supplementary Figure 1	page 9
Supplementary Figure 2	page 10
International Lipidomics Society checklist	page 11

Supplementary materials and methods

Preparation of lipids and proteins for mass spectrometry

Extraction of apolipoprotein E (apoE) - bound lipids

ApoE-bound lipids, isolated by immunoprecipitation, were prepared for mass spectrometric analysis by single-phase extraction using 300 µl cold methanol[1] containing 2.4 pg/µl of the heavy isotope-labelled internal standard phosphatidyl ethanolamine, PtdEtn(C15:0/18:1)-d7 (Avanti Polar lipids, 791638). Samples were thereafter shaken at 2000 rpm for 30 minutes followed by sonication for 15 minutes. The supernatant was separated from the beads by centrifugation at 4 °C, 16 900 x g for 20 minutes and 250 µl of it was carefully pipetted off and transferred to new 1.5-ml microcentrifuge tubes (Sarstedt, 72.690.001). The beads were saved for proteomic analyses. Methanol was evaporated using a vacuum concentrator (Eppendorf).

Prior to instrumental analysis, the samples were reconstituted in 65 µl methanol and transferred to LC micro vials (Thermo Scientific, Chromacol 03-FISV).

Digestion and solid phase extraction of peptides for proteomic analyses

To the already lipid-extracted immunoprecipitated beads, 20 µl digest buffer was added (6 M urea, 2 M thiourea, 2% ASB14, and 200 mM Tris at pH 7.8), containing 3.75 ng/µl whole protein ENO1 (yeast enolase). The samples were shaken at 1500 rpm for 60 minutes. 45 µg dithioerythritol was added to break disulphide bonds, and the samples were shaken again at 1500 rpm for 60 minutes. To prevent these bonds from reforming, 108 µg iodoacetamide was added and the samples were protected from light and shaken at 1500 rpm for 45 minutes. Thereafter, 165 µl MilliQ water was added to dilute the concentration of urea, followed by the addition of 1 µg of LCMS grade trypsin (Promega) for digestion of the proteins into peptides. The samples were incubated at 37 °C for 16 hours. Solid phase extraction was performed to purify and enrich the peptides, using BondElut C18 96-well plates (Agilent). The C18 wells were washed with two 1 ml aliquots of 60% acetonitrile, 0.1% trifluoroacetic acid (TFA) and then equilibrated with two 1 ml aliquots of 0.1% TFA. The samples were adjusted to a 0.1% concentration of TFA and added to the C18 wells. The C18-bound peptides were washed with two 1 ml aliquots of 0.1% TFA and thereafter eluted by two 0.25 ml aliquots of 60% acetonitrile, 0.1% TFA. Solvents were evaporated using a vacuum concentrator.

Instrumental analysis – conditions and settings

Mass spectrometric analysis of lipids

The mobile phase consisted of A: 95% acetonitrile, 5% water with 10 mM ammonium acetate, and B: 50% acetonitrile, 50% water with 10 mM ammonium acetate. The gradient elution profile lasted for 7.75 minutes and was initially set to 100% A for 0.5 minutes, then linearly decreased to 20% A over 3.25 minutes. The column was washed with 100% B for 2 minutes, then set to equilibrate at 100% A for 2 minutes before the subsequent injection. The flow rate was 0.5 ml/min.

The source temperature was 150 °C, the cone gas flow was 150 l/min, and the desolvation gas consisted of nitrogen at a flow rate of 1000 l/h, with a temperature of 650 °C. The argon collision gas was set to a flow rate of 0.25 ml/min. To ensure adequate dwell times, four injections were performed per sample. A quality control sample, created from pooled equal volumes of all samples, was injected every fourth sample to assess instrumental stability.

Targeted apoE analysis by mass spectrometry

The mobile phase consisted of A: water with 0.1% formic acid, and B: acetonitrile, with 0.1% formic acid. The gradient elution profile lasted for 5 minutes and was initially set to 3% B for 0.2 minutes, then linearly increased to 40% B over 3.3 minutes. The column was washed with 99.9% B for 0.75 minutes, then set to equilibrate at 3% B for 0.75 minutes before the subsequent injection. The flow rate was 0.8 ml/min.

The source temperature was 150 °C, the cone gas flow was 150 l/min, and the desolvation gas consisted of nitrogen at a flow rate of 1000 l/h, with a temperature of 500 °C. The argon collision gas was set to a flow rate of 0.15 ml/min. A pooled quality control sample was injected every fourth sample to assess instrumental stability.

Untargeted proteomics mass spectrometry

The mobile phase consisted of A: grade water with 0.1% formic acid, and B: acetonitrile with 0.1% formic acid. The gradient elution applied started at 3% B and was linearly increased to 40% B over 40 minutes after which it was increased to 85% B over 2 minutes and washed for 2 minutes before returning to initial conditions over 1 minute followed by 15 minutes equilibration before the subsequent injection.

The capillary voltage was set to 3 kV and the source temperature to 100 °C. The cone gas consisted of nitrogen with a flow of 50 l/h, the desolvation temperature was set to 200 °C. The purge and desolvation gases consisted of nitrogen, operated at a flow rate of 600 ml/h and 600 l/h respectively. The gas in the IMS cell was helium with a flow rate of 90 ml/h.

Supplementary discussion

Alzheimer's disease risk and apoE plasmalogen level

For most detected ethanolamine plasmalogen (PlsEtn) molecular species, we found an inverse relationship between their degree of association with apoE and E4 allelic dose (D = 0, 1 or 2) (Fig. 3). Furthermore, this relationship appears linear, as evidenced by inspection of the box-and-whisker plot of total PlsEtn/apoE *vs.* D (Fig. 4A) and by the product moment correlation coefficients (Table 2). Is such a linear relationship compatible with PlsEtn levels having a role in mediating the response to D, where that response is Alzheimer's disease risk?

Despite some disadvantages, Alzheimer's disease risk is usually reported in the literature as odds ratios (OR). One of the largest collections for which these measures are available is the

UK Biobank.[2] Using data from this source, if $(OR)_0$, the *OR* for Alzheimer's disease of apoE3E3 ($D = 0$), is taken as 1, then $(OR)_1$ (for apoE3E4, $D = 1$) is 3.69. This value compares closely with that of 3.68 obtained from an online meta-analysis.[3] The UK Biobank result for apoE4E4 ($D = 2$), $(OR)_2$, is 13.52. Since $3.69^2 = 13.62$ (and $3.68^2 = 13.54$), it follows that:

$$(OR)_2 \cong (OR)_1^2$$

suggesting an exponential relationship between *OR* and *D*:

$$(OR)_0 = (OR)_1^0;\ (OR)_1 = (OR)_1^1;\ (OR)_2 = (OR)_1^2$$

While acknowledging a need for caution extrapolating from data necessarily constrained to just three (*x, y*) coordinates, a general expression for the line on which these three points fall would therefore be:

$$\log(OR) = \beta D$$

which corresponds to a version of the logistic regression equation, with β being its coefficient (= log 3.69 in this instance). Thus, the relationship between *OR* and *D* conforms to one of the mathematical models capable of generating a sigmoidal-shaped curve.[4] Given that ΣPlsEtn/apoE is linearly, albeit inversely, related to *D*, it must also share a semilogarithmic connection with *OR*. Without ascribing any particular molecular mechanism to a potential causal link between PlsEtn levels and Alzheimer's risk, the fact that there is a gradient in the graphical representation of Alzheimer's *OR* vs. ΣPlsEtn/apoE which resembles a biological dose-response curve, in its morphology as well as in its monotonicity, is supportive evidence for such a link. Furthermore, this model is potentially useful as a framework for interpreting future studies with apoE isoforms involving apoE2, hence not consisting solely of E3 and/or E4.

Supplementary references

1. Tokuoka SM, Kita Y, Sato M, Shimizu T, Yatomi Y, Oda Y. Lipid profiles of human serum fractions enhanced with CD9 antibody-immobilized magnetic beads. *Metabolites.* 2022;12:230.

2. Lumsden AL, Mulugeta A, Zhou A, Hyppönen E. Apolipoprotein E (*APOE*) genotype-associated disease risks: a phenome-wide, registry-based, case-control study utilising the UK Biobank. *EBioMedicine*. 2020;59:102954.

3. AlzGene. www.alzgene.org/meta.asp?geneID=83 Accessed 22 March 2024.

4. Ritz C. Toward a unified approach to dose-response modeling in ecotoxicology. *Environ Toxicol Chem*. 2010;29:220-229.

Supplementary Table 1 Ethanolamine plasmalogens - monitored transitions					
**Name**	**Precursor ion [m/z]**	**Product ion *sn*-1 [m/z]**	**Product ion *sn*-2 [m/z]**	**Collision energy *sn*-1 /*sn*-2 [V]**	**Cone voltage [V]**
C16:0/16:0	676.5	364.3	313.3	16/24	76
C16:0/18:0	704.6	364.3	341.3	16/24	76
C16:0/18:1	702.5	364.3	339.3	16/24	76
C16:0/18:2	700.5	364.3	337.3	16/24	76
C16:0/18:3	698.5	364.3	335.3	16/24	76
C16:0/20:4	724.5	364.3	361.3	16/24	76
C16:0/20:5	722.5	364.3	359.3	16/24	76
C16:0/22:4	752.6	364.3	389.3	16/24	76
C16:0/22:5	750.5	364.3	387.3	16/24	76
C16:0/22:6	748.5	364.3	385.3	16/24	76
C16:1/16:0	674.5	362.2	313.3	16/24	76
C16:1/18:0	702.5	362.2	341.3	16/24	76
C16:1/18:1	700.5	362.2	339.3	16/24	76
C16:1/18:2	698.5	362.2	337.3	16/24	76
C16:1/18:3	696.5	362.2	335.3	16/24	76
C16:1/20:4	722.5	362.2	361.3	16/24	76
C16:1/20:5	720.5	362.2	359.3	16/24	76
C16:1/22:4	750.5	362.2	389.3	16/24	76
C16:1/22:5	748.5	362.2	387.3	16/24	76
C16:1/22:6	746.5	362.2	385.3	16/24	76
C18:0/16:0	704.6	392.3	313.3	16/24	76
C18:0/18:0	732.6	392.3	341.3	16/24	76
C18:0/18:1	730.6	392.3	339.3	16/24	76
C18:0/18:2	728.6	392.3	337.3	16/24	76
C18:0/18:3	726.5	392.3	335.3	16/24	76
C18:0/20:4	752.6	392.3	361.3	16/24	76
C18:0/20:5	750.5	392.3	359.3	16/24	76
C18:0/22:4	780.6	392.3	389.3	16/24	76
C18:0/22:5	778.6	392.3	387.3	16/24	76
C18:0/22:6	776.6	392.3	385.3	16/24	76
C18:1/16:0	702.5	390.3	313.3	16/24	76
C18:1/18:0	730.6	390.3	341.3	16/24	76
C18:1/18:1	728.6	390.3	339.3	16/24	76
C18:1/18:2	726.5	390.3	337.3	16/24	76
C18:1/18:3	724.5	390.3	335.3	16/24	76
C18:1/20:4	750.5	390.3	361.3	16/24	76
C18:1/20:5	748.5	390.3	359.3	16/24	76
C18:1/22:4	778.6	390.3	389.3	16/24	76
C18:1/22:5	776.6	390.3	387.3	16/24	76
C18:1/22:6	774.5	390.3	385.3	16/24	76

Supplementary Table 2 Phosphatidyl ethanolamines - monitored transitions				
**Name**	**Precursor ion [m/z]**	**Product ion [m/z]**	**Collision energy [V]**	**Cone voltage [V]**
C15:0/18:1-d7	711.6	570.6	20	30
C34:0	720.6	579.5	20	30
C34:1	718.5	577.5	20	30
C34:2	716.5	575.5	20	30
C34:3	714.5	573.5	20	30
C34:4	712.5	571.5	20	30
C36:0	748.6	607.6	20	30
C36:1	746.6	605.6	20	30
C36:2	744.6	603.5	20	30
C36:3	742.5	601.5	20	30
C36:4	740.5	599.5	20	30
C36:5	738.5	597.5	20	30
C38:0	776.6	635.6	20	30
C38:1	774.6	633.6	20	30
C38:2	772.6	631.6	20	30
C38:3	770.6	629.6	20	30
C38:4	768.6	627.5	20	30
C38:5	766.5	625.5	20	30
C38:6	764.5	623.5	20	30
C38:7	762.5	621.5	20	30
C40:0	804.6	663.6	20	30
C40:1	802.6	661.6	20	30
C40:2	800.6	659.6	20	30
C40:3	798.6	657.6	20	30
C40:4	796.6	655.6	20	30
C40:5	794.6	653.6	20	30
C40:6	792.6	651.5	20	30
C40:7	790.5	649.5	20	30
C40:8	788.5	647.5	20	30

Supplementary Table 3 Apolipoprotein E peptides - monitored transitions

Compound	Amino acid sequence	Precursor ion [m/z]	Product ions [m/z]	Collision energy [V] ion 1/ion 2	Cone voltage [V]
apoE (E3/E4)	LAVYQAGAR	475.1	502.3/665.4	16/16	28
apoE (E4)	LGADMEDVR	503.5	835.5/829.5	16/16	46
apoE (E2)	CLAVYQAGAR	554.8	345.1/835.6	14/14	14
apoE (E2/E3)	LGADMEDVCGR	611.8	491.2/606.3	26/18	56
apoE	AATVGSLAGQPLQER	749.8	642.4/827.6	18/26	54
Enolase 1 (yeast)	GNPTVEVELTTEK	709.1	623.5/948.7	18/20	35

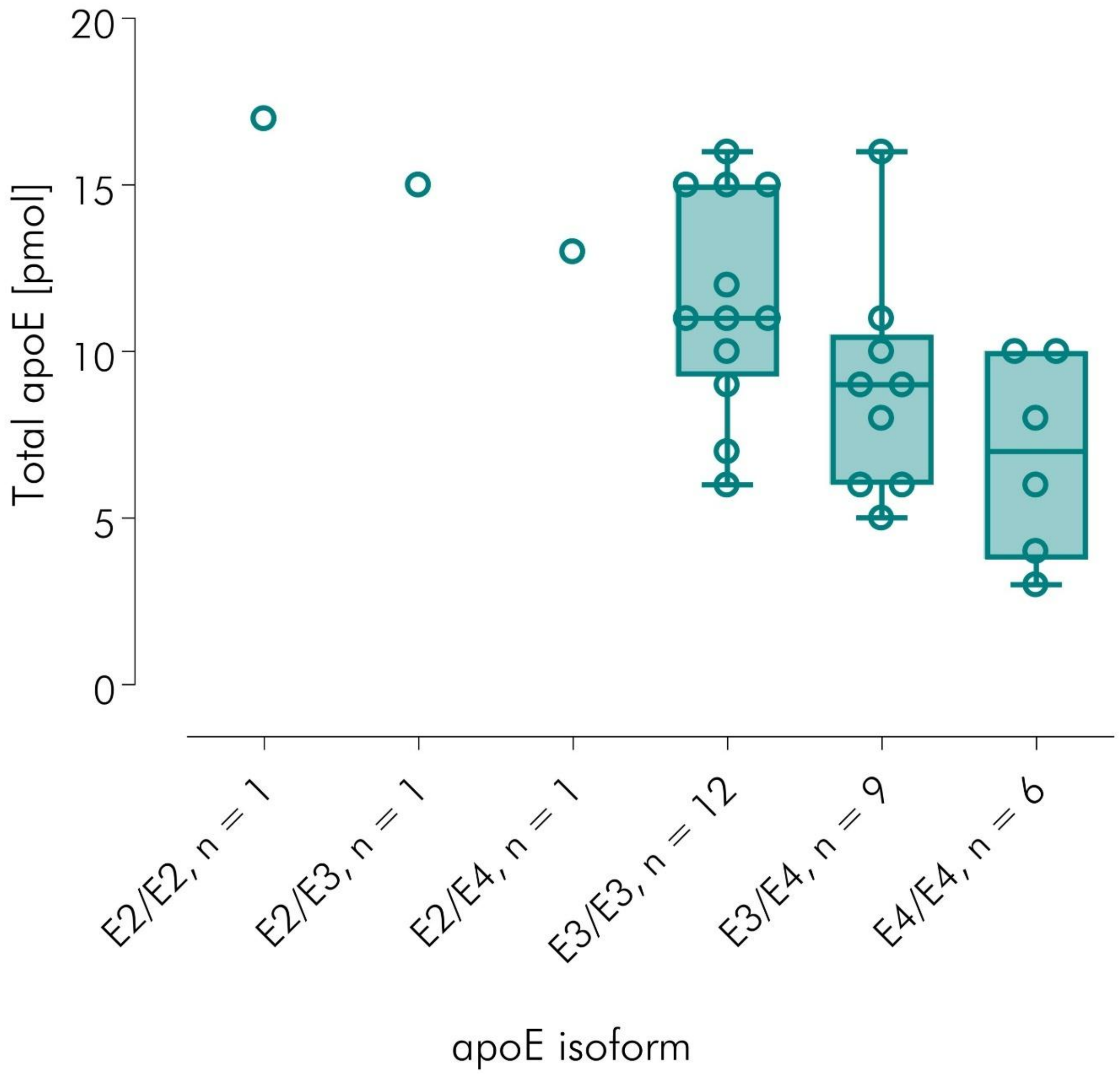

Supplementary Figure 1 Apolipoprotein E (apoE) levels in human CSF according to apoE genotype/isoform. Each data point (open circle) represents a CSF sample from an individual participant. For isoforms where $n > 1$, the apoE levels are shown in box-and-whisker plots where the whiskers show the minimum and maximum values and the boxes show the 25th percentile, the median and the 75th percentile. For these same isoforms (E3E3, E3E4 and E4E4), using single factor ANOVA, an overall *P*-value of 0.0054 was obtained (F = 4.3) but the only inter-group comparison which was statistically significant using Fisher's Least Significant Difference Test was E3E3 *vs.* E4E4 ($P = 0.0019$).

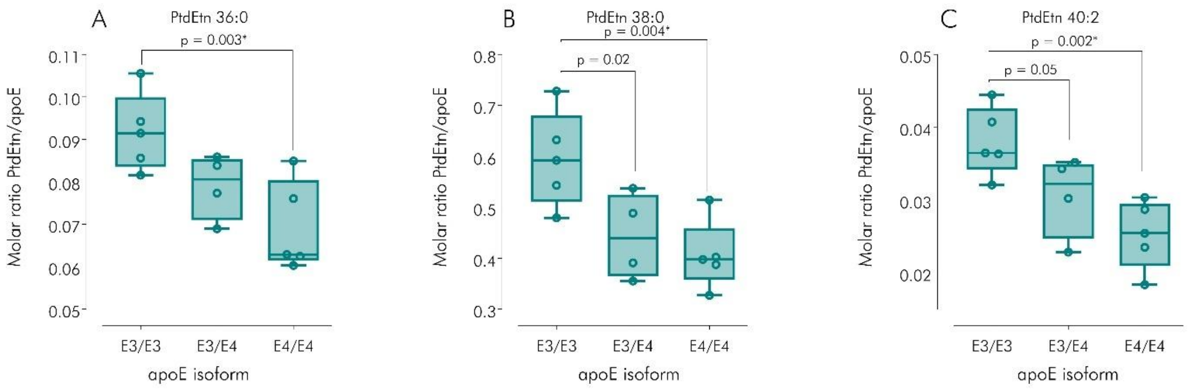

Supplementary Figure 2 Phosphatidylethanolamine-apolipoprotein E molar ratios. (**A** – **C**) Molar ratios of phosphatidylethanolamine (PtdEtn) to apolipoprotein E (apoE), according to apoE isoform (E3E3 n = 5, E3E4 n = 4, or E4E4 n = 5) and to PtdEtn species. Box-and-whisker plots where the whiskers show the minimum and maximum values and the boxes show the 25th percentile, the median and the 75th percentile. Each data point (small open circle) represents a CSF sample from an individual participant. Only the three PtdEtn species illustrated (from a total of 18) showed a significant reduction in PtdEtn content comparing apoE4E4 with apoE3E3 and these did not include the most abundant species (C38:4). Nominal P-values were determined using single factor ANOVA, followed by Fisher's Least Significant Difference test and are shown in the plots, values significant post multiple testing correction (Benjamini-Hochberg procedure, 5% false discovery rate) are annotated by an asterisk (see Materials and Methods).

General Lipidomics Workflow

Created by https://lipidomicstandards.org, version v2.2.0

Overall study design

Title of the study	APOE/Plasmalogen CSF IP study		
Document creation date	04/21/2023	Corresponding Email	l.ginsberg@ucl.ac.uk
Principle investigator	Lionel Ginsberg and Kevin Mills	Is the workflow targeted or untargeted?	Targeted
Institution	UCL GOS ICH	Clinical	No

Lipid extraction

Extraction method	1-phase system	1-phase system	Methanol
pH adjustment	None	Were internal standards added prior extraction?	Yes

Analytical platform

Number of separation dimensions	One dimension	MS vendor	Waters
Separation Type 1	LC	Ion source	ESI
Separation Mode 1	NP	MS Level	MS1, MS2
Separation window (1) for lipid analyte selection (±) in minutes	1	Mass resolution for detected ion at MS1	Low resolution
RT verified by standard	Yes	Resolution in Da at MS1	0.1
CCS verified by standard	Yes	Mass window for precursor ion isolation (in Da total isolation window)	0
Separation of isobaric/isomeric interferece confirmed	Yes	Mass resolution for detected ion at MS2	Low resolution
Model for separation prediction	No	Resolution in Da at MS2	0.1
MS type	QQQ	Was/Were additional dimension/techniques used	No

Quality control

Blanks	Yes	Quality control	Yes
Type of Blanks	Extraction blank, Injection blank	Type of QC sample	Sample pool

Method qualification and validation

Method validation	No

Reporting

Are reported raw data uploaded into repository?	Available on request	Additional comments	-
Raw data upload	Available on request		

Sample Descriptions

APOE IP CSF / Human / Other liquid material

Provided information	-	Additives	None
Temperature handling original sample	4-8 °C	Were samples stored under inert gas?	No
Instant sample preparation	Yes	Additional preservation methods	No
Storage temperature	-80 °C	Biobank samples	No

Lipid Class Descriptions

1) PE[M+H]+ / Lipid identification

Lipid class	PE	Check isomer overlap	No
MS Level for identification	-	Additional dimension/techniques	-
Identification level	Species level	Lipid Identification Software	Homemade
Polarity mode	Positive	Data manipulation	Smoothing
Type of positive (precursor)ion	[M+H]+	Nomenclature for intact lipid molecule	Yes

1) PE[M+H]+ / For additional separation methods/analytical dimension

Quantitative	Yes	Type I isotope correction	No
MS Level for quantification	-	Limit of quantification	S/N ratio
Type of quantification	Calibration line	Normalization to reference	No
Type of calibration line	Solvent based	Lipid Quantification Software	Homemade
Species calibration line	PE mixture (Matreya, 1069).	Batch correction	No
Response correction	No		

2) PE O-p[M+H]+ / Lipid identification

Lipid class	PE O-p	Check isomer overlap	No
MS Level for identification	-	Additional dimension/techniques	-
Identification level	sn Position	Lipid Identification Software	Homemade
Polarity mode	Positive	Data manipulation	Smoothing
Type of positive (precursor)ion	[M+H]+	Nomenclature for intact lipid molecule	Yes

2) PE O-p[M+H]+ / For additional separation methods/analytical dimension

Quantitative	Yes	Type I isotope correction	No
MS Level for quantification	-	Limit of quantification	S/N ratio
Type of quantification	Calibration line	Normalization to reference	No
Type of calibration line	Solvent based	Lipid Quantification Software	Homemade
Species calibration line	PE mixture (Matreya, 1069) and PE O-P 18:0/18:1 (Avanti Polar lipids, 85275P)	Batch correction	No
Response correction	No		
